# Vascular smooth muscle cell phenotypic transition regulates gap junctions of cardiomyocyte

**DOI:** 10.1007/s00380-020-01602-3

**Published:** 2020-04-08

**Authors:** En Zhou, Tiantian Zhang, Changlong Bi, Changqian Wang, Zongqi Zhang

**Affiliations:** 1 Department of Cardiology, Shanghai Ninth People’s Hospital, School of Medicine, Shanghai Jiao Tong University, 639 Zhizaoju Road, Shanghai, 200011 China; 2 Shanghai Jiao Tong University School of Medicine, 227 South Chongqing Road, Shanghai, 200025 China

**Keywords:** Connexin, Smooth muscle cell, Cardiomyocyte, Phenotypic transition, MicroRNA 27b

## Abstract

Atrial fibrillation (AF) is one of the most prevalent arrhythmias. Myocardial sleeves of the pulmonary vein are critical in the occurrence of AF. Our study aims to investigate the effect of synthetic vascular smooth muscle cells (SMCs) on gap junction proteins in cardiomyocytes. (1) Extraction of vascular SMCs from the pulmonary veins of Norway rats. TGF-β_1_ was used to induce the vascular SMCs switching to the synthetic phenotype and 18-α-GA was used to inhibit gap junctions of SMCs. The contractile and synthetic phenotype vascular SMCs were cocultured with HL-1 cells; (2) Western blotting was used to detect the expression of Cx43, Cx40 and Cx45 in HL-1 cells, and RT-PCR to test microRNA 27b in vascular SMCs or in HL-1 cells; (3) Lucifer yellow dye transfer experiment was used to detect the function of gap junctions. (1) TGF- β_1_ induced the vascular SMCs switching to synthetic phenotype; (2) Cx43 was significantly increased, and Cx40 and Cx45 were decreased in HL-1 cocultured with synthetic SMCs; (3) The fluorescence intensity of Lucifer yellow was higher in HL-1 cocultured with synthetic SMCs than that in the cells cocultured with contractile SMCs, which was inhibited by18-α-GA; (4) the expression of microRNA 27b was increased in HL-1 cocultured with synthetic SMCs, which was attenuated markedly by 18-α-GA. (5) the expression of ZFHX3 was decreased in HL-1 cocultured with synthetic SMCs, which was reversed by 18-α-GA. The gap junction proteins of HL-1 were regulated by pulmonary venous SMCs undergoing phenotypic transition in this study, accompanied with the up-regulation of microRNA 27b and the down-regulation of ZFHX3 in HL-1 cells, which was associated with heterocellular gap junctions between HL-1 and pulmonary venous SMCs.

## Introduction

Atrial fibrillation (AF) is one of the most prevalent arrhythmias, which increased the risk of heart failure, stroke and sudden death [1, 2]. It has been well established that paroxysmal AF is mainly triggered by cardiomyocytes located in the pulmonary veins [3], and these cells are characterized by electrical remodeling such as delayed electrical conduction and short refractory period [4].

Gap junctions consisting of proteins from the connexin (Cx) family play a crucial role in the electrical remodeling of cardiomyocytes like intercellular ion transfer [5] to contribute to the pathogenesis of AF. For examples, Cx43 was downregulated by JNK activation promoting AF development [6], and Cx40 was related to idiopathic AF clinically [7]. In atrium, it mainly expresses Cx40, Cx43 and Cx45. In the pulmonary vein, connexins expression and molecular properties of ion channels have been demonstrated to resemble those of the working myocardium under physiological conditions [8], such as Cx43 in the pulmonary sleeves comparable to atrial myocardium [9]. However, when under pathological conditions, connexins remodeling occurring in pulmonary vein could play a pivotal role in AF initiation [10], such as Cx40 protein that was downregulated markedly in the pulmonary sleeves of AF dogs [10]. However, the regulation of connexins expression in pulmonary venous cardiomyocytes is not fully elucidated.

Accumulating evidence has demonstrated that the neighboring cells could regulate gap junctions of cardiomyocytes. In the heart chamber, cardiomyocytes neighbor with fibroblasts or myofibroblasts. Cx43 in the cardiomyocytes could be augmented by myofibroblasts not fibroblasts to form heterocellular gap junctions with myofibroblasts [11]. The heterocellular gap junctions would allow slow electronic conduction across scar tissue to induce a reentrant circle [12], which was also considered as an important mechanism of AF initiation.

However, the cardiomyocytes in pulmonary sleeves were surrounded by the vascular smooth muscle cells (SMCs) [13], which provides an anatomical basis for the establishment of gap junctions between cardiomyocytes and SMCs. In hypertension, the most common risk factor of AF, the pulmonary venous SMCs had an increase of α-SMA expression and collagen deposition, showing that SMCs switched to the synthetic phenotype. Meanwhile, AF or atrial tachycardia was induced more frequently by pacing [14]. Furthermore, when AF occurs, it has been revealed that the expression of Cx40 was attenuated and Cx43 was augmented markedly in pulmonary venous cardiomyocytes [8, 10]. Therefore we hypothesize that SMCs with synthetic phenotype may affect the expression of connexin proteins in pulmonary venous cardiomyocytes, and induce heterocellular gap junctions between these two types of cells in the pulmonary vein.

There is an emerging role of microRNA (miRNA) in connexins regulation of cardiomyocytes [15, 16]. MiRNAs are short nucleotide sequences that bind to the 3′-untranslated region of mRNA, thereby regulating gene expression at the post-transcriptional level by inhibiting the translation of a protein or by promoting mRNA degradation. Recently, miR-1 downregulation was demonstrated to increase Cx43 in cardiomyocytes to promote ventricular arrhythmias [17]. MiR-27b was reported to regulate Cx40 expression in cardiomyocytes of fat mice and then to increase vulnerability to atrial arrhythmia [18]. And miR-130a decreased Cx43 in cardiomyocytes resulting in both atrial and ventricular arrhythmias [16]. However, the roles of miRNAs from SMCs in the regulation of gap junction remodeling of cardiomyocytes remains unknown.

This study aims to investigate the effects of synthetic SMCs on gap junctions of cardiomyocytes and the roles of microRNAs from SMCs in gap junction remodeling of cardiomyocytes.

## Methods

## Ethical approval

All animal experiments were approved by the Animal Research Ethics Committee of the No. 9 People’s hospital affiliated to Shanghai Jiaotong University School of Medicine. All animals were killed by bilateral thoracotomy operation during anesthesia.

### Isolation of pulmonary venous SMCs

Two-month-old male Norway rats were purchased from Shanghai SLAC Laboratory Animal Co. (Shanghai, China). The vascular SMCs were isolated from the pulmonary veins of Norway rats as described previously [19]. The rat was anesthetized with chloral hydrate and was then subjected to thoracotomy. The pulmonary vein was clamped at both ends and was excised. Then the vein was washed twice in physiological saline and was put into DMEM/high glucose media (HyClone; GE Healthcare Life Sciences, Utah, USA; Cat#SH30243.01; Lot#AB216032) containing 10% fetal calf serum (gibco; Rochester, NY, USA; Cat#10099-141; Lot#2059461RP). To obtain vascular SMCs with high purity, the intima and adventitia of vein were completely removed. The medial layer of vein was cut into small pieces and attached to a cell culture dish. After 4–7 days, vascular SMCs would crawl out of the tissue pieces, followed by the digestion and purification using differential adherence.

### Phenotypic switching of SMCs

To examine the effect of synthetic vascular SMCs on gap junctions in cardiomyocytes, we first used transforming growth factor beta 1 (TGF-β_1_) to induce phenotypic switching of vascular SMCs. Vascular SMCs were cultured in a 6-wells plate at 5 × 10^4^ per well. After adherence, vascular SMCs were stimulated by TGF-β_1_ 5 ng/ml; (Millipore; Billerica, MA, USA; Cat#GF439, Lot#VP1801100) to induce the phenotypic transition. After 24 h, the culture medium was discarded and the cells were washed three times with phosphate buffer saline (PBS) prepared for the following experiments.

### Immunocytochemistry

SMCs cultured on membranes were rinsed with PBS, fixed for 15 min at room temperature, blocked for 2 h in PBS containing 5% normal goat serum and 2% BSA, and incubated with monoclonal antibodies (diluted 1:1000) against α-actin (Santa Cruz Biotechnology; Dallas, Texas, USA; Cat#sc-32251; Lot#B0615; RRID:AB_262054) at 4 °C overnight. Cells were rinsed five times in PBS and incubated with Alexa Fluor 594-conjugated (red) goat anti-mouse antibody (diluted 1:500) for 1 h at room temperature.

### Inhibition of gap junction

SMCs were stimulated by TGF-β_1_, followed by the treatment with 50 μM 18-α-glycyrrhetinic (18-α-GA) (Sigma; Cat#G8503) dissolved in 0.5% DMSO for 24 h to block the formation of the gap junction. Cells were treated by DMSO were used as control. HL-1 cells alone and HL-1 cells cocultured with SMC in coculture system treated with 50 μM 18-α-GA dissolved in 0.5% DMSO for 24 h to detect the toxic effect of 18-α-GA.

### Coculture of vascular SMCs and cardiomyocytes

HL-1 cell line was purchased from Yuanchuang Biotechnology Co., Ltd (Shanghai, China). HL-1 cells and SMCs were cultured in DMEM/high glucose media containing 10% fetal calf serum at 37 °C in a humidified 5% CO_2_ incubator and used after 3–4 passages. The 24 mm-Transwell Inserts with 0.4 μm pores (Sigma Cat#CLS3450-24E) were used to establish the coculture system. In contact coculture system, the vascular SMCs were seeded on the bottom side of 6-well Transwell insert membranes at the concentration of 5 × 10^4^ cells/membrane. And after 6 h, HL-1 cells were seeded on the top side of 6-well Transwell insert membranes. In non-contact coculture system, the vascular SMCs were seeded on the bottom of 6-well at the concentration of 5 × 10^4^ cells/well. After 6 h, Hl-1 cells were seeded on the top side of 6-well Transwell insert membranes. After HL-1 was grown over the entire insert, all cells were collected separately for real-time PCR and Western blotting.

### Real-time PCR

Total RNA was isolated from HL-1 or vascular SMCs separately from the two side of the Transwell insert membranes using TRIzol reagent (ambion by life technologies, Carlsbad, CA, USA; Cat#10296028; Lot#79348502) and followed by specific steps with the manufacturer’s protocol. RNA was reverse-transcribed to cDNA using the Thermo Cycler machine (Applied Biosystems). qRT-PCR was performed using a Real-time PCR machine (LightCycler 480 II, Roche). The possible target genes mRNA was quantified using Takara reverse transcription assay (Takara Cat#R0037A) and SYGRII (Takara Cat#RR820A). Specific primers were synthesized from Sangon Biotech (Shanghai, CHINA). The mRNA levels were quantified with the 2^−ΔΔct^ (Table 1).

**Table 1:** The sequences of the primers

Gene	Forward 5′ → 3′	Reverse 5′ → 3′
Collagen I	TGTTGGTCCTGCTGGCAAGAATG	GTCACCTTGTTCGCCTGTCTCAC
Vimentin	GTCCGTGTCCTCGTCCTCCTAC	TAGAGGCTGCGGCTAGTGCTG
Calponin	ACCACCAGCGTGAGCAGGAG	CGTCCTTGAGGCCATCCATGAAG
Mir27b	GCGAGAGCTTAGCTGATTGGT	AGTGCAGGGTCCGAGGTATT
GAPDH	GACATGCCGCCTGGAGAAAC	AGCCCAGGATGCCCTTTAGT

### Western blotting

To investigate the effect of vascular SMCs on gap junction proteins in cardiomyocytes, HL-1 cells were cocultured with vascular SMCs using transwell system. Western blotting was used to detect related gap junction proteins in HL-1 cells. Total protein of HL-1 cells or SMCs was extracted by RIPA separately from the two side of the Transwell insert membranes and separated by 10% SDS-PAGE, electrophoretically transferred to PVDF membranes, and probed with the following primary antibodies including anti-Cx43 (Cell Signaling Technology, Danvers, MA, USA; Cat#3512, Lot#4, RRID:AB_2294590), anti-Cx45 (Abcam; Discovery Drive, Cambridge Biomedical Campus, Cambridge, CB2 0AX, UK; Cat#ab78408, Lot#GR3187454-1, RRID:AB_1566083), anti-Cx40 (Santa Cruz Biotechnology Cat#sc-365107, Lot#C2417, RRID:AB_10708736), anti-α-tubulin (Cell Signaling Technology Cat#2144, Lot#0005, RRID:AB_2210548) in 4 ℃ overnight. After that, the membranes were incubated by horseradish peroxidase (HRP)-conjugated secondary antibodies in room temperature for 1 h. Analysis was conducted using the ECL system (Fusion FX7).

### Dye transfer

After receiving the stimulation with TGF-β_1_ or then treated by 18-α-GA, vascular SMCs were loaded with lucifer yellow biocytin (5 mg/mL, Thermo Fisher Scientific Cat#L-6950), using a pinocytotic uptake method (Invitrogen, Grand Island, NY; Cat#I14402). Then HL-1 cells were cocultured with vascular SMCs at 5 × 10^4^ per well that had received the stimulation with TGF-β_1_ or 18-α-GA or not. After 24 and 48 h, lucifer yellow biocytin in vascular SMCs or HL-1 cells was observed as green fluorescence at the excitation/emission wavelengths of 428 nm/536 nm as described previously [20]. cTNT antibody was used to label HL-1 cells and incubated with Alexa Fluor 594-conjugated (red) goat anti-mouse antibody. The mean fluorescence intensity of Lucifer yellow was analyzed by ImageJ software (ImageJ, RRID:SCR_003070) [21].

### Statistical analysis

Mean ± standard error was used to represent the quantitative data, which was analyzed by ANOVA followed by least significant difference (LSD) *t* test for post-hoc comparison using the SPSS 13.0 software(SPSS,RRID:SCR_002865). *P* values < 0.05 were considered statistically significant.

## Results

### *TGF-β*_*1*_* induces phenotypic switching of vascular SMCs*

**Fig. 1:**
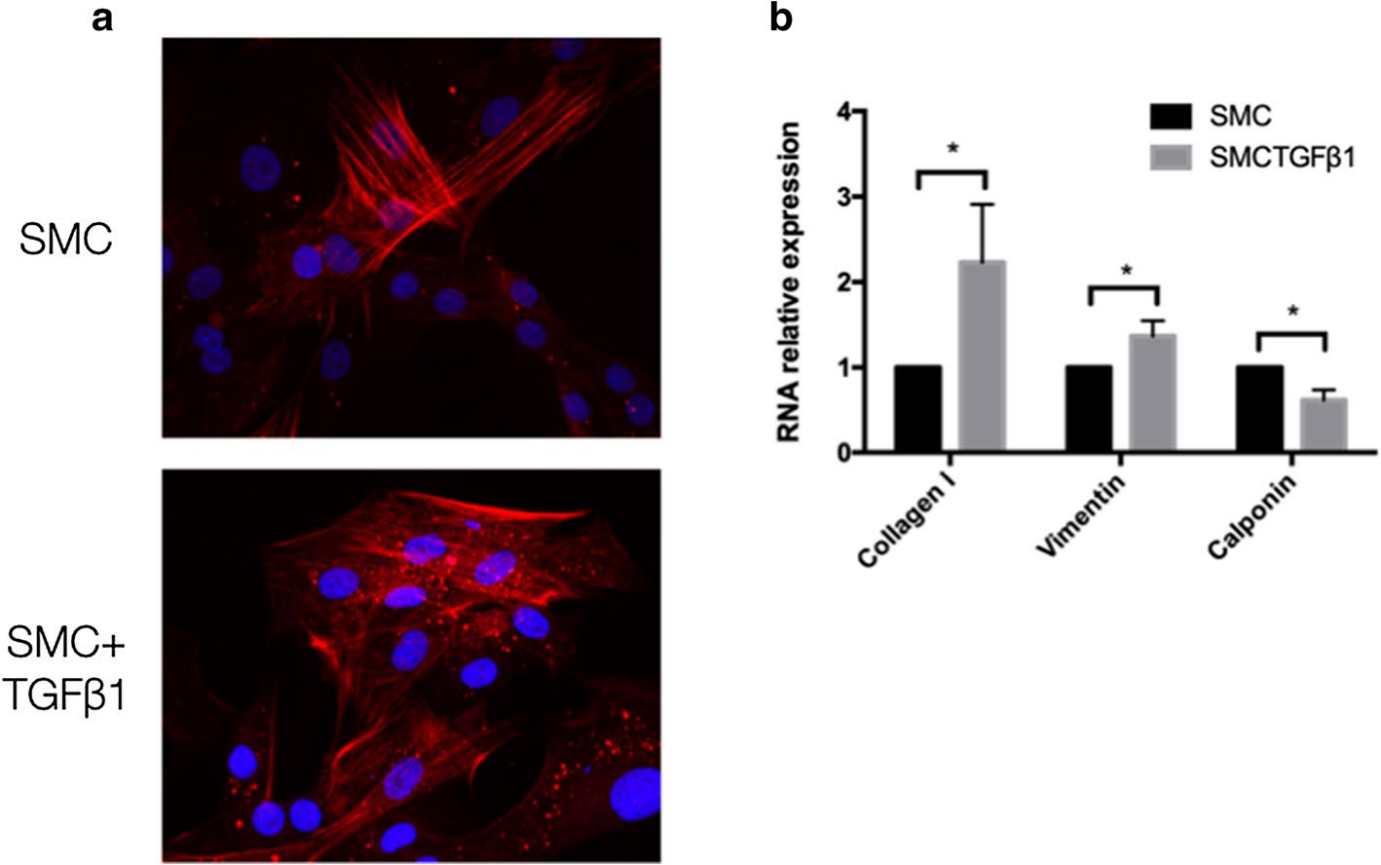
Phenotypic switching of SMCs. **a**. Immunofluorescence staining of a-SMA in SMCs receiving the treatment of TGF-β_1_ (5 ng/mL) or not; **b** the mRNA levels for markers of contractile and synthetic SMCs were detected by RT-PCR (*n* = 5). *P < 0.05, *SMC* smooth muscle cell, *SMC + TGFβ*_*1*_ smooth muscle cell treated by TGF-β_1_ (5 ng/mL)

Vascular SMCs treated with TGF-β_1_ showed a typical "valley-peak" growth pattern and diffuse actin staining (Fig. 1a). The mRNA expressions of col1agen I and vimentin, the markers of vascular SMCs synthetic phenotype, were significantly increased. At the same time, calponin, a marker of contractile phenotype, was decreased markedly (Fig. 1b).

### Vascular SMCs with synthetic phenotype regulate the expression of connexins in cardiomyocytes

**Fig. 2:**
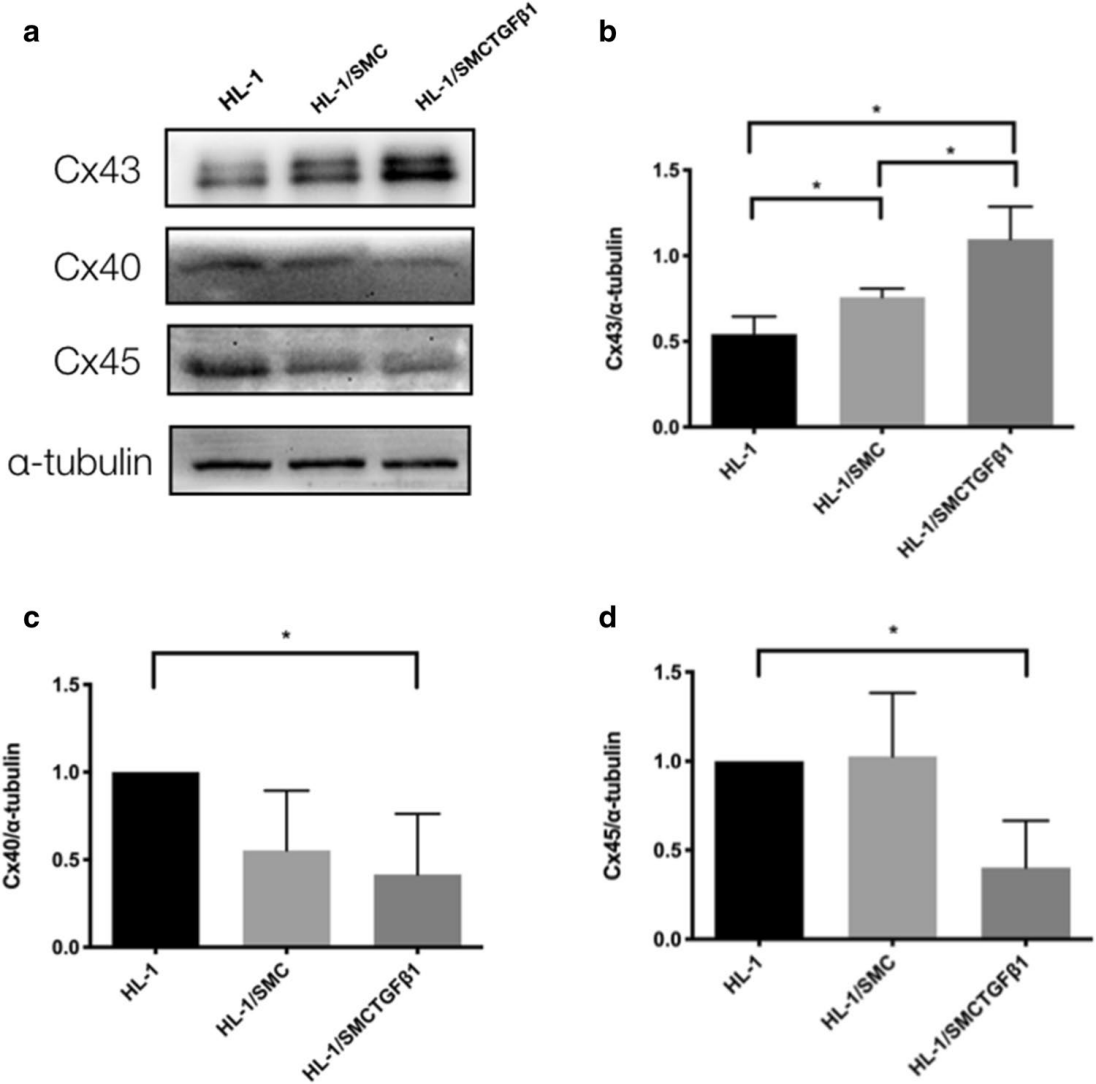
Effects of SMCs on gap junction proteins expression in HL-1 cells. Cx43, Cx40 and Cx45 were detected using western blot (**a**), and the relative protein levels of these molecules were determined by densitometric analysis (**b**–**d**) (*n* = 5). Data are shown as mean ± SD. *, *P* < 0.05. *Cx43* connexin43, *Cx40* connexin40, *Cx45* connexin45, *HL-1* HL-1 cells, *HL-1/SMC* HL-1 cells which were cocultured with contractile SMCs for 48 h, *HL-1/SMC TGFβ*_*1*_ HL-1 cells which were cocultured with synthetic SMCs for 48 h. The number of observations (*n*) represents the number of independent cell preparations

**Fig. 3:**
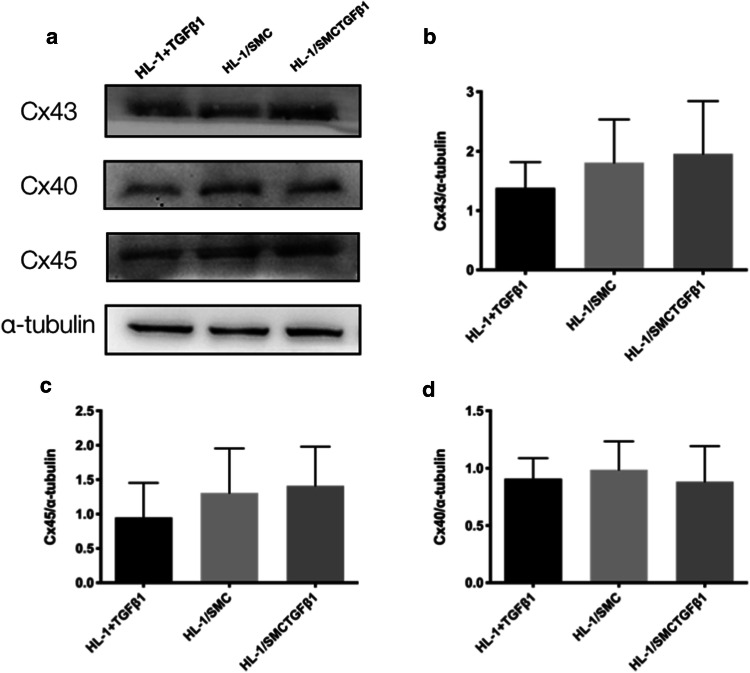
Expression of gap junction proteins in HL-1 cells in non-contact coculture model. Cx43, Cx40 and Cx45 were detected using western blot (**a**), and the relative protein levels of these molecules were determined by densitometric analysis (**b**–**d**) (*n* = 5). Data are shown as mean ± SD. *, *P* < 0.05. *Cx43* connexin43, *Cx40* connexin40, *Cx45* connexin45, *HL-1 + TGFβ*_*1*_ HL-1 cells induced by TGFβ_1_, *HL-1/SMC* HL-1 cells cocultured with SMCs for 48 h in non-contact coculture system, *HL-1/SMCTGFβ*_*1*_ HL-1 cells cocultured with synthetic SMCs for 48 h in non-contact coculture system. The number of observations (*n*) represents the number of independent cell preparations

HL-1 cells had an initial level of Cx43, Cx40 or Cx45 (Fig. 2a). Cx43 expression in HL-1 cells was increased significantly when cocultured with SMCs and was elevated further when cocultured with SMCs treated by TGF-β_1_ (Fig. 2b). Meanwhile, the expressions of Cx40 and Cx45 were notably decreased in HL-1cells cocultured with SMCs treated by TGF-β_1_ (Fig. 2c, d). However, in the shared media coculture system, the expressions of Cx43, Cx40 and Cx45 were not changed in HL-1 cells cocultured separately with SMCs whether treated with TGF-β_1_ or not (Fig. 3).

### Vascular SMCs with synthetic phenotype form functional gap junctions with cardiomyocytes

**Fig. 4:**
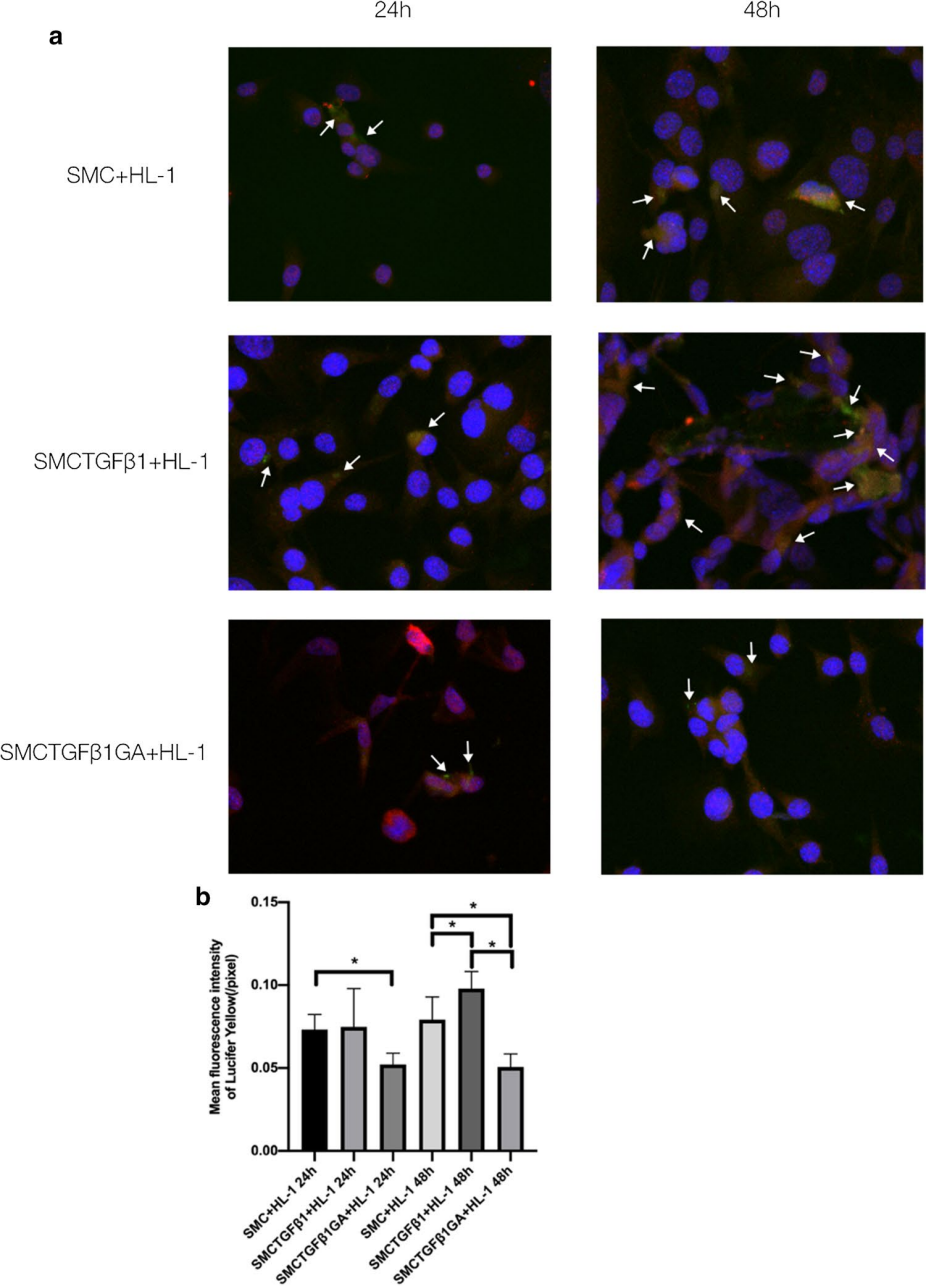
Heterocelluar gap junctions between SMCs and HL-1. Gap junctions were detected using lucifer yellow biocytin transfer. Brilliant green fluorescence of lucifer yellow biocytin in HL-1 cells (red) was showed by yellow fluorescence, indicating biocytin transfer from SMCs to HL-1 cells (white arrowheads in **a**; × 400). **b** Semi-quantitative assessment of lucifer yellow biocytin transfer to HL-1 cells by pixel intensity (mean ± SD). *SMC + HL-1* HL-1 cells cocultured with contractile SMCs, *SMCTGFβ*_*1*_* + HL-1* HL-1 cells cocultured with synthetic SMCs, *SMCTGFβ*_*1*_*GA + HL-1* HL-1 cells cocultured with synthetic SMCs which were treated with 18-α-GA. The data are presented as the mean ± SD of four independent experiments and analyzed by LSD *t* test

After 48- not 24-h coculture with SMCs treated by TGF-β_1_, Lucifer yellow transferred from SMCs into HL-1 cells, as evidenced by cytoplasmic green fluorescence markedly increased in HL-1 cells. However, such an increase was attenuated significantly by 18-α-GA administrated to synthetic SMCs beforehand. In addition, cytoplasmic green fluorescence was hardly detected in HL-1 cells cocultured with the contractile-like SMCs (Fig. 4).

### Vascular SMCs with synthetic phenotype increase miR-27b in HL-1 cells

**Fig. 5:**
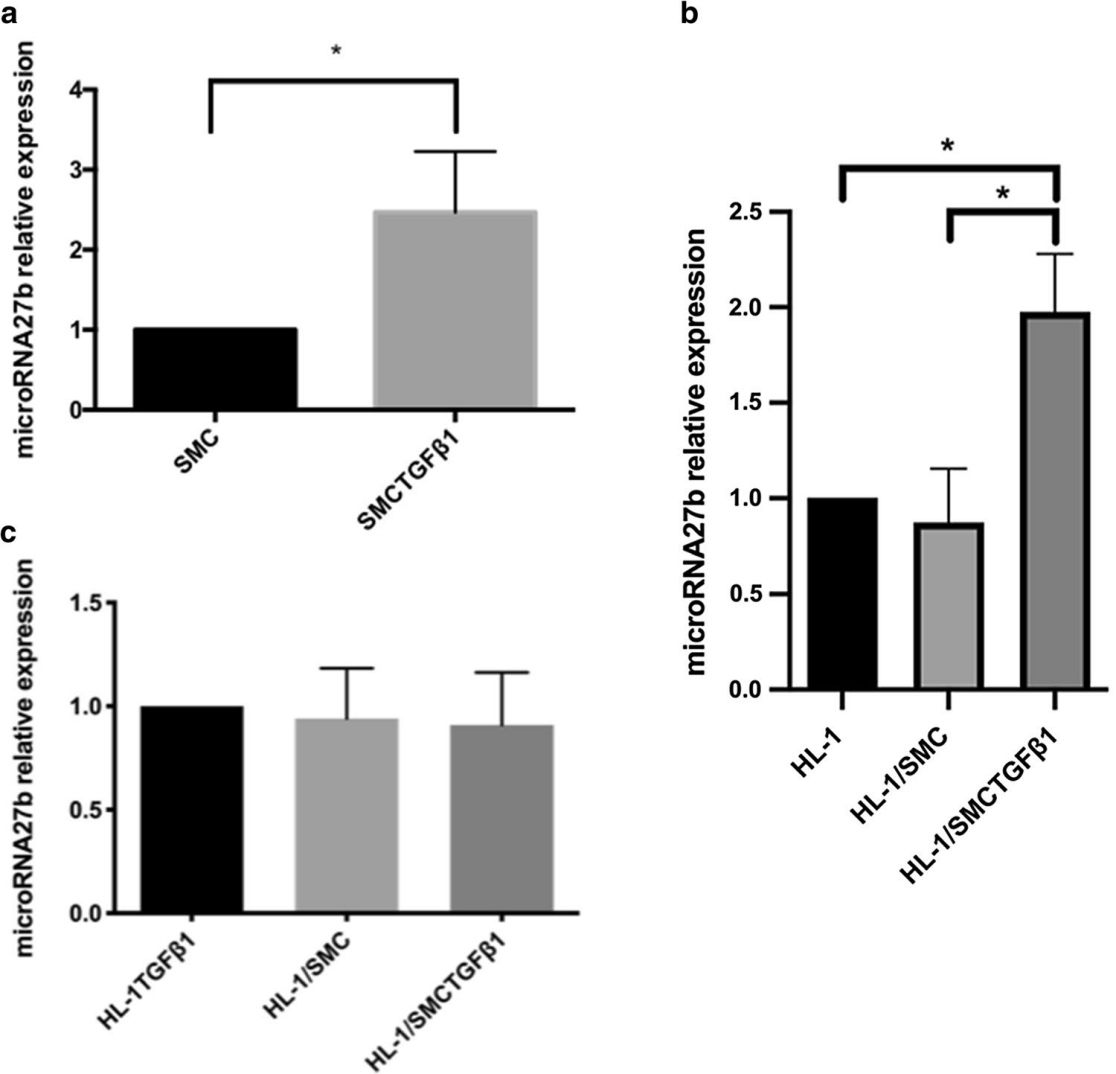
MiR-27b expression in SMCs and HL-1 cells. The expression of miR-27b was detected by RT-PCR. **a** miR-27b expression in contractile SMCs and in synthetic SMCs (*n* = 5); **b** miR-27b expression in HL-1 cells cocultured with SMCs for 48 h (*n* = 5). *HL-1* HL-1 cells, *HL-1/SMC* HL-1 cells which were cocultured with contractile SMCs, *HL-1/SMCTGFβ*_*1*_ HL-1 cells which were cocultured with synthetic SMCs. **c** miR-27b expression in HL-1 cells cultured in non-contact coculture system (*n* = 5). *SMC* contractile SMCs, *SMCTGFβ*_*1*_ synthetic SMCs, *HL-1TGFβ*_*1*_ HL-1 cells treated by TGFβ_1_, *HL-1/SMC* HL-1 cells cocultured with SMCs in non-contact coculture system, *Hl-1/SMCTGFβ*_*1*_ HL-1 cells cocultured with synthetic in non-contact coculture system. *P < 0.05

The expression of miR-27b was significantly increased in HL-1 cells cocultured with SMCs with synthetic phenotype not with contractile-like SMCs (Fig. 5a). At the same time, the expression of miR-27b was increased about 2.4 times in SMCs treated with TGFβ_1_ compared to that in normal SMCs (Fig. 5b). In non-contact coculture system, the expression of miR-27b of HL-1 cells was comparable in three groups (Fig. 5c).

### Heterocellular gap junctions are involved in miR-27b and ZFHX3 expressions in HL-1 cells

**Fig. 6:**
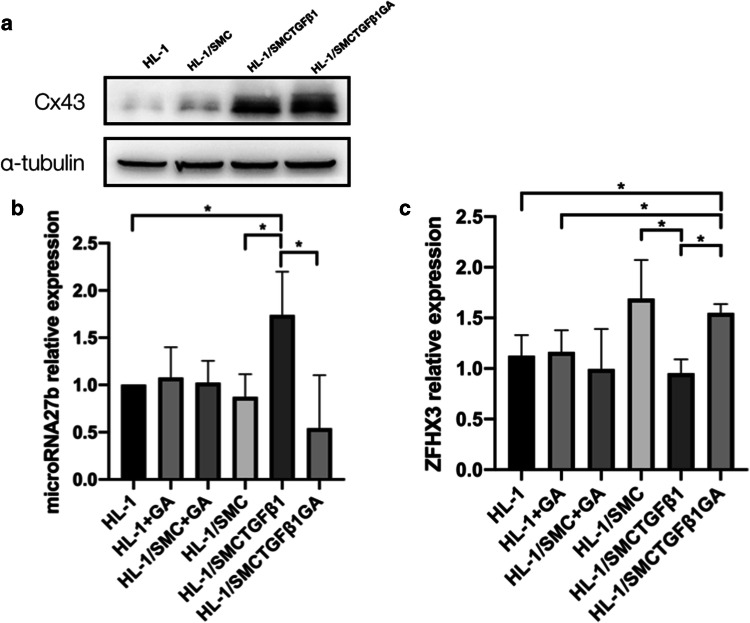
The effects of 18-α-GA on the expression of Cx43, miR-27b and ZFHX3 in HL-1 cells. The expression of Cx43 was detected by Western blotting. The expression of miR-27b and ZFHX3 was detected by RT-PCR. **a** The expression of Cx43 in HL-1 cells cocultured with SMCs for 48 h (*n* = 5). **b** miR-27b expression in HL-1 cells cocultured with SMCs for 48 h (*n* = 5). **c** The ZFHX3 expression in HL-1 cells cocultured with SMCs for 48 h (*n* = 5). *HL-1* HL-1 cells, *HL-1 + GA* HL-1 cells treated with 18-α-GA for 24 h, *HL-1/SMC + GA* HL-1 cells cocultured with SMC then treated with 18-α-GA for 24 h, *HL-1/SMC* HL-1 cells which were cocultured with contractile SMCs, *HL-1/SMCTGFβ*_*1*_ HL-1 cells which were cocultured with synthetic SMCs, *HL-1/SMCTGFβ*_*1*_*GA* HL-1 cells which were cocultured with synthetic SMCs treated by 18-α-GA for 24 h. *P < 0.05

18-α-GA, which was used to inhibit gap junctions of synthetic SMCs, did not change the expression of Cx43 in HL-1 cells (Fig. 6b). However, the increased miR-27b in HL-1 cells was decreased to 0.5 times when being cocultured with synthetic SMCs treated by 18-α-GA in advance (Fig. 6b). Meanwhile, the expression of ZFHX3 was decreased in HL-1 cells coculture with synthetic SMCs, which was reversed by 18-α-GA treated to synthetic SMCs forehead (Fig. 6c). Furthermore, 18-α-GA treated both in HL-1 group (HL-1 + GA) and HL-1/SMC group (HL-1/SMC + GA). The expression of miR-27b and ZFHX3 in these two group was comparable to the HL-1 group (Fig. 6b, c).

## Discussion

Atrial fibrillation is one of the most prevalent arrhythmias, and to make clear the pathogenesis of atrial fibrillation is very important. In this study, we found that the gap junctions in HL-1 cells were regulated by synthetic pulmonary venous SMCs, accompanied with the increased miR-27b and decreased ZFHX3 markedly in HL-1 cells.

Pulmonary vein is surrounded by an external sleeve of cardiomyocytes that are widely recognized as triggers of paroxysmal atrial fibrillation. In the pulmonary vein, the cardiomyocytes were localized surrounding the vascular SMCs. Accumulating evidence has well demonstrated that the architecture of pulmonary venous sleeves is associated with the possibility of initiating AF [22]. For instance, thicker or longer sleeves were more frequent AF [23]. Recently, it was reported that more synthetic-like SMCs presenting in the pulmonary vein of hypertensive rats were more likely subjected to AF [14, 24]. However, it remains to be elucidated whether these synthetic-like SMCs directly regulate bioactivities of cardiomyocytes.

In hypertensive patients, the circulating transforming growth factor-β_1_ (TGF-β_1_) is significantly increased [25], a pivotal factor to induce the phenotypic transition of contractile-like SMCs to synthetic-like cells [26]. In this study, we used TGF-β_1_ to induce SMCs with synthetic phenotype as evidenced by the notable diffusing actin, increased collagen I and vimentin, and decreased calponin as described in our previous study [27].

Connexins, gap junction proteins, have been widely proved to play an important role in physiological electronic conduction of cardiomyocytes [28]. Cx43 expresses in both atrium and ventricle. In this study, SMCs were cocultured with HL-1 cells using a transwell system to anatomically mimic these cells in pulmonary venous sleeves. We found that Cx43 was significantly increased in HL-1 cells cocultured with synthetic pulmonary venous SMCs. The current findings suggested that phenotypic transition of pulmonary venous SMCs, as previously described in myocardial sleeves [14], might change Cx43 expression in cardiomyocytes, leading to the abnormal electrical conduction between SMCs and cardiomyocytes to promote AF initiation.

It was notable that ventricular Cx43 reduction (e.g., induced by JNK activation) would lead to heart dysfunction linking to an enhanced propensity to AF [29–31], however, increasing cardiac gap junctional intercellular communication by ZP123 failed to attenuate atrial tachyarrhythmias inducibility [32]. Taken together, these results suggest the different roles of Cx43 expression in atrial or ventricular cardiomyocytes in AF initiation.

Besides the increase of Cx43, we found a marked decrease of Cx40 and Cx45 in HL-1 cells when cocultured with synthetic pulmonary venous SMCs. Previous studies showed that Cx40 expression was lower in myocardial sleeves than that in left atrium, and was further downregulated in the myocardial sleeve of AF dogs [9, 10], which were consistent to our current findings. Previous study found Cx45 labeled strongly in the human myocardial sleeves [3]. Taken together, these results suggested that the decrease of Cx40 and Cx45 in pulmonary venous sleeves in AF development, which might be concerned with the phenotypic switching of pulmonary venous SMCs.

Previous study has shown that the cellular expression of gap junction proteins could be regulated by paracrinal cytokines, which could be also secreted from synthetic SMCs [33]. However, in the present study, the expression of Cx43, Cx40 or Cx45 in HL-1 cells was little affected by the synthetic SMCs separately cocultured with HL-1 cells in the non-contact coculture system, suggesting that the changed expressions of Cx43, Cx40 and Cx45 did not result from synthetic SMCs in a paracrinal manner.

Intercellular gap junctions were closely affected by connexins regulation. Changed connexins expression could make the formation of heterocellular gap junction between cardiomyocytes and non-cardiomyocytes to contribute to arrhythmias [34, 35]. In this study, intercellular communication (biocytin transfer) was detected in HL-1 cells cocultured with synthetic SMCs not contractile-like SMCs, suggesting the intercellular communication was fully formed probably due to increase of Cx43 expression in both these cells, similar to the previous results of Cx43-dependent gap junctions between cardiomyocytes and myofibroblasts [36].

Gap junctions are intercellular channels to allow passage of ions and small molecules to achieve cell-specific delivery including miRNAs [37]. MiR-27b was recently reported to be positively related to vulnerability to AF initiation [18]. In this study, we found that miR-27b was upregulated in the synthetic pulmonary venous SMCs. Furthermore, miR-27b was significantly increased in HL-1 cells cocultured with these synthetic SMCs in contact coculture system, which was markedly inhibited by 18-α-GA administrated to synthetic SMCs forehead, without change of Cx43 expression in HL-1 cells. These findings suggested that the increased miR-27b in HL-1 cells might be transferred from synthetic SMCs cocultured with HL-1 cells through heterocellular gap junctions.

Mir-27b has been reported to target several genes contributing to AF, such as ZFHX3 [38]. For examples, ZFHX3 knock-down may cause dysregulated calcium homeostasis and increased atrial arrhythmogenesis [39] and cause cardiac remodeling [40], contributing to AF development. In this study, the expression of ZFHX3 was significantly decreased in HL-1 cocultured with synthetic SMCs, accompanied by an increase of miR-27b. In addition, 18-α-GA, a nonselective inhibitor of gap junctions administrated to synthetic SMCs, could reverse the declination of ZFHX3 in HL-1 cells. And the toxic effect of 18-α-GA has little effect on the expression of miR-27b and ZFHX3 in HL-1 cells. In this study, the transferred lucifer yellow biocytin was detected little in HL-1 cells cocultured with contractile-like SMCs, suggesting that HL-1 cells did not fully form heterocellular gap junctions with the contractile-like SMCs. These results might account for the little effect of 18-α-GA on miR-27b expression in HL-1 cells cocultured with the contractile-like SMCs. In addition, there is an increase of ZFHX3 expression in HL-1 cells, even not statistically significant, which was caused by 18-α-GA treated to the contractile-like SMCs. It was noticeable that ZFHX3 was a target gene of miR-1 [41], which was reported to be negatively regulated by long non-coding RNA MALAT1 through gap junction connexins such as Cx43 [42]. Then it needs to be explored further whether the inhibition of heterocellular gap junctions by 18-α-GA in SMCs could negatively regulate ZFHX3 expression via the MALAT1-Cx43-miR-1 axis in HL-1 cells. These results suggested that the heterocellular gap junctions were involved in the down-regulation of ZFHX3 in HL-1 cells, which might be due to the increase of miR-27b in these cells.

### Limitations

Although the changed expression of gap junction proteins in the cardiomyocytes cocultured with SMCs undergoing phenotypic switching was observed, these results were based on experiments in vitro, which needs to be elucidated further in large animals in vivo. Although our results implied that miR-27b affected little the expressions of Cx43 and Cx40 in HL-1 cells, the specific mechanism of the changed expression of gap junction proteins needs to be further explored.

In the present study, biocytin was detected in HL-1 cells cocultured with contractile SMCs, in spite of little, suggesting that HL-1 constructed heterocellular gap junctions with contractile SMCs. However, it remains to be explored which connexin was involved in the full formation of gap junctions.

Although our results suggested that the increase of miR-27b contributed to the changed ZFHX3 in HL-1 cells, it needs to be confirmed whether ZFHX3 is the direct target gene of miR-27b in HL-1 cells. It also needs further study to explore whether the inhibition of heterocellular gap junctions by 18-α-GA in SMCs could negatively regulate ZFHX3 expression via the MALAT1-Cx43-miR-1 axis in HL-1 cells.

In summary, the gap junctions of HL-1 was affected by the pulmonary venous SMCs undergoing phenotypic transition in this study, accompanied by the up-regulation of miR- 27b and the down-regulation of ZFHX3 in HL-1 cells, which could be reversed markedly by 18-α-GA, a non-selective inhibitor of gap junctions, administrated to synthetic SMCs. The current findings suggested that the phenotypic switching of pulmonary venous SMCs could regulate ZFHX3 gene expression of cardiomyocytes, associated with functionally heterocellular gap junctions between these two types of cells in pulmonary venous sleeves, which might contribute to AF initiation.
